# *RPE65*-associated inherited retinal diseases: consensus recommendations for eligibility to gene therapy

**DOI:** 10.1186/s13023-021-01868-4

**Published:** 2021-06-04

**Authors:** Andrea Sodi, Sandro Banfi, Francesco Testa, Michele Della Corte, Ilaria Passerini, Elisabetta Pelo, Settimio Rossi, Francesca Simonelli, Italian IRD Working Group

**Affiliations:** 1 Department of Ophthalmology, Careggi Teaching Hospital, Florence, Italy; 2 Medical Genetics, Department of Precision Medicine, University of Campania “Luigi Vanvitelli”, Naples, Italy; 3 Telethon Institute of Genetics and Medicine (TIGEM), Pozzuoli, NA Italy; 4 Eye Clinic, Multidisciplinary Department of Medical, Surgical and Dental Sciences, University of Campania “Luigi Vanvitelli”, Via S. Pansini, 5, 80131 Naples, Italy; 5 Department of Genetic Diagnosis, Careggi Teaching Hospital, Florence, Italy

**Keywords:** Gene therapy, Inherited retinal diseases, *RPE65*, Voretigene neparvovec

## Abstract

**Background:**

This research aimed to establish recommendations on the clinical and genetic characteristics necessary to confirm patient eligibility for gene supplementation with voretigene neparvovec.

**Methods:**

An expert steering committee comprising an interdisciplinary panel of Italian experts in the three fields of medical specialisation involved in the management of *RPE65*-associated inherited retinal disease (IRD) (medical retina, genetics, vitreoretinal surgery) proposed clinical questions necessary to determine the correct identification of patients with the disease, determine the fundamental clinical and genetics tests to reach the correct diagnosis and to evaluate the urgency to treat patients eligible to receive treatment with voretigene neparvovec. Supported by an extensive review of the literature, a series of statements were developed and refined to prepare precisely constructed questionnaires that were circulated among an external panel of experts comprising ophthalmologists (retina specialists, vitreoretinal surgeons) and geneticists with extensive experience in IRDs in Italy in a two-round Delphi process.

**Results:**

The categories addressed in the questionnaires included clinical manifestations of *RPE65*-related IRD, IRD screening and diagnosis, gene testing and genotyping, ocular gene therapy for IRDs, patient eligibility and prioritisation and surgical issues. Response rates by the survey participants were over 90% for the majority of items in both Delphi rounds. The steering committee developed the key consensus recommendations on each category that came from the two Delphi rounds into a simple and linear diagnostic algorithm designed to illustrate the patient pathway leading from the patient’s referral centre to the retinal specialist centre.

**Conclusions:**

Consensus guidelines were developed to guide paediatricians and general ophthalmologists to arrive at the correct diagnosis of *RPE65*-associated IRD and make informed clinical decisions regarding eligibility for a gene therapy approach to *RPE65*-associated IRD. The guidelines aim to ensure the best outcome for the patient, based on expert opinion, the published literature, and practical experience in the field of IRDs.

**Supplementary Information:**

The online version contains supplementary material available at 10.1186/s13023-021-01868-4.

## Background

Inherited retinal diseases (inherited retinal dystrophies; IRDs) are a heterogeneous group of ocular neurodegenerative disorders resulting from mutations in any one of over 250 causative genes [1]. They are mostly characterised by progressive retinal degeneration that leads to severe visual impairment and blindness [2–6]. Inherited retinal dystrophy caused by confirmed biallelic mutations in the *RPE65* gene, which encodes all-trans retinyl ester isomerase, an enzyme critical to the visual cycle, is a serious and sight-threatening autosomal recessive genetic disorder that causes a severe form of rod-cone mediated IRD that eventually progresses to complete blindness [3–5]. The spectrum of *RPE65*-mediated IRD exhibits common clinical findings, initially characterised by nyctalopia (night blindness), present from early childhood and due to a primary effect on the rod photoreceptors [7, 8]. The visual function of individuals affected with IRD declines with age, with deteriorating visual acuity (VA) and progressive loss of retinal structure and function (retinal sensitivity) on visual field testing by Goldmann kinetic perimetry (GVF), often leading to blindness in young adulthood [8–11]. The disease course may include earlier or later onset, nystagmus, along with night blindness and loss of vision. Indeed, individuals with biallelic *RPE65* mutations may be given one of a variety of clinical diagnoses. Depending on the time of disease onset, severity, rate of progression and presenting phenotype, the most common diagnoses are Leber congenital amaurosis (LCA) and early-onset severe retinal dystrophy (EOSRD) [5, 8]. These forms are thought to be responsible for approximately 5% of cases of severe IRDs [7]. However, a smaller proportion of patients exhibit a milder phenotype with a slower progression, possibly associated with hypomorphic alleles [11–13].

Initially considered incurable, as the understanding of the pathophysiological mechanisms underlying the subtypes of IRD has expanded, a number of therapeutic approaches to treating IRDs have been proposed, the most advanced of which is gene supplementation therapy [6]. Monogenic ocular diseases are good candidates for gene transfer therapy, as the eye has favourable anatomical and immunological characteristics, providing a contained physical space protected by the blood-ocular barrier that is particularly suited for local delivery [14]. Remarkably, *RPE65*-associated IRD represents a successful model for the development of ocular gene supplementation therapy applied to monogenic diseases.

The proof of principle of gene therapy for *RPE65*-associated IRD was demonstrated in murine and canine models of LCA [15–17], in which a recombinant adeno-associated viral vector serotype 2 (AAV2) gene replacement therapy produced encouraging improvements in visual function. This led to a clinical trials programme that confirmed the safety, durable efficacy, and favourable benefit-to-risk profile of voretigene neparvovec (AAV2-h*RPE65*v2, voretigene neparvovec-rzyl, LUXTURNA™; Spark Therapeutics, Inc, Philadelphia, PA, USA, Novartis, Basel, Switzerland), administered as a (one-time) sub-retinal injection, in improving retinal and visual function in *RPE65*-mediated IRD [18–30].Voretigene neparvovec received marketing authorisation for the US in 2017 [31] and the European Union in 2018 [32] for the treatment of adult and paediatric patients with vision loss due to IRD related to confirmed *RPE65* biallelic mutations and who have sufficient viable retinal cells [32].

A precise genetic diagnosis is necessary to establish eligibility for treatment of *RPE65*-associated IRD and to optimise the use of a precision therapeutic intervention such as voretigene neparvovec in a clinically and genetically heterogeneous group of IRDs. Not only is there a lack of shared criteria for the selection of patients suitable for *RPE65* gene therapy, but the cost and complexity of the procedure mean that an equitable and transparent process for evaluating the urgency to treat for eligible patients is also necessary. In the absence of specific national guidance in this area, the goal of this project was to develop a clinical pathway algorithm that sets forth a stepwise process for ophthalmologists and geneticists to make decisions about the correct diagnosis and treatment with voretigene neparvovec of patients with *RPE65*-associated IRD. Herein, we report the outcomes of a consensus process by a group of Italian experts in IRDs to establish recommendations on the clinical and genetic characteristics necessary to confirm patient eligibility for gene therapy with voretigene neparvovec.

## Methods

A steering committee (the authors of this paper) comprising an interdisciplinary panel of Italian experts in the three fields of medical specialisation involved in the management of *RPE65*-associated IRD (medical retina, genetics, vitreoretinal surgery) was established to investigate the correct identification of patients with the disease, identify the fundamental clinical tests to determine the correct diagnosis and to evaluate the urgency to treat patients eligible to receive treatment with voretigene neparvovec. The Delphi technique is a recognised and reliable means of consensus-building utilising a series of precisely constructed questionnaires to collect data from an external survey panel with recognised experience in the field of interest [33, 34]. A standard Delphi process was chosen to reach consensus among a wider external panel of experts comprising ophthalmologists (chosen from the applicable sub-specialties of retina specialists, paediatric ophthalmologists and vitreoretinal surgeons) and geneticists with extensive experience in IRDs in Italy who were identified and invited to participate in the survey rounds. The survey participants, named as the Italian IRD Working Group and listed in Additional file 6: Appendix, were carefully selected to minimise the risk that, in such a multidisciplinary field, there could be skills gaps between the respondents.

### Delphi process

The extensive clinical expertise of the steering committee was drawn upon to build broad and open-ended statements for the first Delphi questionnaire in order to elicit further qualitative input on the diagnostic and treatment approaches of the participating experts to be considered for inclusion in a second Delphi round. The initial questionnaire was supported by a comprehensive review of the literature to identify the current understanding of the disease and the place of voretigene neparvovec gene supplementation in its treatment [35]. The categories covered in the literature review included: *RPE65*-related IRD, IRD screening and diagnosis, gene testing and genotyping, ocular gene therapy for IRDs, and voretigene neparvovec. The candidate items were finalised to ensure that they were understandable and exactly captured the information needed for designing the decision pathway. Then, they were reviewed by a small, independent validation panel of experts comprising one expert from each specialty (genetics, ophthalmology) and not part of the population surveyed (see Additional file 6: Appendix 1) before the questionnaire was distributed for administration to the wider survey panel.

A 5-point Likert scale in which 1 = *strongly disagree*, 2 = *disagree*, 3 = *neither agree nor disagree*, 4 = *agree* and 5 = *strongly agree* was proposed to rate the answers generated in the Delphi rounds. Disagreement was defined as a Likert response of 1, 2 or 3; Agreement as a Likert response of ≥ 4, and Consensus was defined as a level of agreement of ≥ 70%. Although a universally-agreed proportion has not been established for the Delphi process [36], a 70% cut-off was chosen as a rigorous way of determining consensus, with agreement from over two-thirds of the panel considered to provide a reliable indicator of consensus. The stability of the data was tested by assessing the change in the degree of consensus between Round 1 and Round 2 [33].

As part of the questionnaire format, potential challenges relating to the multidisciplinary nature of the survey population were addressed by further analysing the responses through stratification of the sample according to specialty (clinical, genetic, surgical). Taken together with the participants’ self-assessed degree of confidence in the specific sub-topic, section by section, a more precise interpretation of the questionnaire responses was facilitated.

### Refinement of Round 1 results

The results of the Round 1 questionnaire were further developed by the steering committee, considering all of the important differences in responses among the medical specialties, and modifying or re-proposing statements that did not achieve consensus in Round 1. The second-round questionnaire, therefore, built on the statements in each section and incorporated additional statements designed to capture the range of information necessary to establish the diagnostic algorithm. The revised statements were circulated electronically and underwent a second Delphi consensus round. The Round 2 survey results were then ordered and prioritised towards the development of the diagnostic algorithm designed to refine the selection of patients and optimise treatment outcomes.

## Results

### Key consensus recommendations

Response rates by the survey participants were over 90% for the majority of items in both Delphi rounds. Key consensus recommendations from the two Delphi rounds are summarised in Table 1 and the consensus findings of the Round 1 and Round 2 questionnaires are detailed in Additional files 1, 2, 3, 4 and 5. The following is a summary of the consensus of the indications obtained in the course of the Delphi approach.

**Table 1:** Summary of the key consensus recommendations regarding anamnesis and genetic testing

*Anamnesis*
When assessing a patient with suspected *RPE65* mutation-associated inherited retinal disease, the anamnesis (medical history) must include:
General ophthalmologic history
Symptoms at onset
Age at symptom onset
Pedigree
Inquiry about consanguinity
Existence of other affected family members
Signs of disease progression
Previous/current therapy for ocular diseases
Other previous visits to a general ophthalmologist or to a retina specialist
Previous clinical assessment for vision (MRI, OCT, ERG, FAF)
Presence or not of neurological or extra-ocular symptoms
General pharmacologic history and ongoing medical treatments
History of infectious diseases
Other diseases
Patient expectations
Professional activities and lifestyle (for adult patients)
*Genetic testing*
Genetic testing for diagnosis must be carried out by certified laboratories
The certification of a genetic diagnostic laboratory is defined by the following criteria
ISO certification
Analysis of > 100 cases per year for genetic diagnosis and document a highly significant number of confirmed genetic diagnosis cases
Being part of a network with medical geneticists and inherited retinal diseases specialists from other national and international centres
The certified laboratories conducting genetic testing for inherited retinal disease diagnosis must
Have qualified geneticists with consolidated expertise in the genetics of hereditary retinal dystrophies
Have standardised internal molecular analysis protocols
Perform genetic counselling before and after testing
Be part of a national diagnostic laboratories network and/or Genetic Scientific Society (e.g., SIGU)
Rely on a complete multidisciplinary team (geneticists, retina specialist, molecular biologists, technicians, bioinformatician, genetic counsellor) already familiar with IRDs molecular diagnosis
Be able to perform MLPA analysis
Be able to perform both Sanger and multi-gene NGS tests
Be able to perform in silico analysis
Be able to perform in vitro protein functional assessment
Participate in inherited retinal disease national/international registries
A qualified geneticist is defined as a geneticist with
Consolidated expertise in the genetics of hereditary retinal dystrophies
Updated knowledge of the state-of-the-art and proven track record in the field of genetics of IRDs
Relevant published literature in the field
Proactive interactions and collaborations with international counterparts as part of multicentre consortia
Active networking with national and international counterparts is particularly important for a qualified geneticist
In the case of rare diseases with high genetic heterogeneity like *RPE65*-associated Inherited retinal disease
To exchange knowledge and expertise with other geneticists and IRD specialists

*ERG* electroretinography, *FAF* fundus autofluorescence, *IRD* inherited retinal disease, *MLPA* multiplex ligation-dependent probe amplification, *MRI* magnetic resonance imaging, *GS* next generation sequencing, *OCT* optical coherence tomography, *SIGU* Società Italiana Genetica Umana (the Italian Society of Human Genetics)

### Clinical Manifestations of ***RPE65***-associated IRD (Additional file 1: Table S1)

1. When assessing a patient with suspected *RPE65*-associated IRD, the anamnesis (medical history) must include:
  Inquiry about consanguinity, the existence of other affected family members, history of infectious diseases and other diseases, and pedigree.
  The taking of general ophthalmological history, symptoms at onset, age at symptom onset, other previous visits to a general ophthalmologist or to a retina specialist, previous ophthalmological clinical assessment (see fundamental clinical diagnostic tests below), signs of disease progression previous, and current therapy for ocular diseases.
  Presence or absence of neurological or extra-ocular symptoms, general pharmacological history and ongoing medical treatments, patient expectations, professional activities and lifestyle (for adult patients).
2. The role of clinical diagnosis in reaching a level of suspicion that justifies genetic testing in the presence of a hereditary retinal dystrophy was emphasised. *RPE65*-associated IRD should be suspected in individuals with the following clinical findings:
  Symptomatic onset between birth and 5 years, nystagmus or roving eye movements, profound nyctalopia and decreased central VA.
  Fundus appearance that tends to be normal in infancy and then ranging from RPE mottling to pigmentary retinopathy with attenuated vessels and optic nerve pallor [37].
  Full-field electroretinogram (ERG) barely detectable or severely abnormal.
  Severely diminished or absent fundus autofluorescence (FAF) and a relatively preserved central retinal structure at optical coherence tomography (OCT) [38].
3. The order of clinical manifestations should be undertaken to give them priorities and weights depending on their frequency in patients and to characterise their importance for diagnosis. Night blindness, narrowing of the visual field and reduced VA are the three symptoms that are present; in infants, light staring (photoattraction) is characteristic of *RPE65*- or *LRAT*-associated IRDs. Nystagmus is often associated with this condition, but not all patients have it.
4. The clinical diagnosis is mainly LCA or EOSRD/EORP. Nevertheless, *RPE65* biallelic mutations can be also associated with Fundus albipunctatus (FA) a rare form of stationary night blindness [39].
5. Visual acuity may vary and is heterogeneous between patients at the onset. Generally, central VA is worse when the onset is before age 1 year compared with onset between 1 and 5 years of age.
6. Fundus examination can be quite variable and can appear normal at presentation.
7. Other fundus findings include RPE mottling, white dots at the level of the RPE, pigmentary retinopathy with attenuated vessels and optic nerve pallor.
8. The basic clinical diagnostic tests to guide towards a level of suspicion that justifies genetic testing (i.e. tests with the highest diagnostic value) are fundus examination, full-field electroretinogram (ERG), GVF and OCT. Tests for further investigation should include FAF, full-field light sensitivity threshold (FST) testing and microperimetry.

### Order of testing for viable retinal cells (Additional file 2: Table S2)

The clinical tests able to assess viable retinal cells in order of priority are the following: (1) OCT, followed by (2) standard ophthalmological examinations including VA, posterior segment biomicroscopy, then (3) GVF testing, microperimetry, colour picture, FAF and full-field ERG.

### Diagnostic genetic testing: Sanger sequencing versus targeted, multi-gene NGS panel (Additional file 3: Table S3)

1. Genetic testing must be prescribed and performed in all cases where there is suspicion of an inherited retinopathy. Genetic testing must be carried out by a certified laboratory affiliated with medical geneticists and inherited retinal diseases specialists from other national and international centres.
2. A strong consensus was reached on the utility of a next-generation sequencing (NGS) approach for the genetic diagnosis of potential *RPE65*-associated IRD cases over a Sanger approach limited to the analysis of the *RPE65* gene. The consensus recommendation was to use a targeted multi-gene NGS approach, including all the genes known to be responsible for IRDs, both isolated and syndromic forms. The use of a larger panel (i.e. either a clinical exome or a whole-exome sequencing) is not excluded but, due to the issue of possible incidental findings, requires a more careful pre-test counselling.
3. Regarding segregation analysis, the issue of collecting a sample from the parents was identified and segregation defined as a necessary step.
4. The issue of variants of uncertain significance (VUS) is important. Genotypes including pathogenic and likely pathogenic variants constitute a straightforward eligibility indication for the treatment. However, genotypes, including VUS, provide more problems in that respect [40–46]. The panel agreed that they should not be excluded altogether but evaluated on a case-by-case basis. Among additional criteria that can possibly be considered when VUS arise:
  The extension of the NGS panel used for diagnosis (example: a clinical exome or a whole-exome only pointing to an *RPE65* genotype and not in other IRD genes);
  The availability of a negative comparative genomic hybridisation (cGH) array test;
  The results of in silico prediction studies to aid in evaluating “wild type” and mutated molecular models of the *RPE65* protein; and
  The possibility of performing functional in vitro mutagenesis with functional protein assays.
  The presence of a clinical phenotype compatible with *RPE65* mutation.

### Patient eligibility and prioritisation (Additional file 4: Table S4)

1. In general, it is important to give priority to the treatment of paediatric patients, in order to provide them with an opportunity for maintaining better VA (as they are likely to be in a less advanced stage of the disease) and enjoying normal social growth. In accordance with the current level of evidence from clinical trials, a paediatric patient should be considered as a candidate for gene therapy with voretigene neparvovec starting from the age of 3 years. Commencing treatment even earlier may provide greater benefit, and can be expected to increase as more data become available.
2. Shared precise criteria for admission to this treatment has not yet been established. At present, we may refer to the inclusion criteria of the approval studies. More specifically, in the phase 3 study (ClinicalTrials.gov Identifier: NCT00999609), eligibility for inclusion included VA of 20/60 or worse and/or less than 20 degrees of residual GVF in any meridian. Sufficient retinal cell viability, defined as an area of retina within the posterior pole of > 100 μm thickness, was assessed by means of OCT [28].
3. Age is not a criterion for exclusion from gene therapy in patients with age over 3 years.
4. Early treatment for patients with good VA is recommended to prevent progression. Conversely, low VA is not a criterion for exclusion from gene therapy because it may help to preserve the remaining vision. In Italy, treatment is reimbursed only for a VA of 0.5 LogMar or lower.
5. Psychological assessment, patient’s attitude towards treatment and compliance to follow-up assessments post-treatment are important.
6. Although not yet considered fully reliable as a prognostic marker, monitoring the rate of disease progression using multimodal imaging [47] can be considered a useful criterion for prioritising patients eligible to receive treatment with voretigene neparvovec.

### Consideration of surgical issues (Additional file 5: Table S5)

1. On the basis of a comprehensive anamnesis suggestive of *RPE65*-associated IRD, patients must be referred to a clinical centre where ophthalmologists are specialised in the diagnosis of IRDs and have the ability to prescribe IRD genetic testing and genetic counselling from a certified laboratory with consolidated expertise in the genetics of IRD.
2. On confirmation of eligibility to voretigene neparvovec gene therapy by an IRD-specialised centre, the procedure must be carried out in a paediatric hospital setting that provides children-centred care appropriate for children aged 3–6 years and that is able to deliver and properly manage paediatric anaesthesiology procedures.
3. The vitreoretinal surgeon who performs the subretinal injection must have surgical experience with paediatric and adult patients with IRD and must be affiliated to an IRD centre certified for the use of gene therapy with voretigene neparvovec.
4. To corroborate patient eligibility for surgery and to assess the risks of subretinal injection, the retina specialist must discuss with the certified surgeon the expectations of the patient regarding the clinical outcome of the procedure while considering the age of the patient, retinal thickness, the presence of any other eye disorders, eligibility for general anaesthesia and an evaluation of the risk/benefit ratio of treatment.
5. The best surgical strategy for each patient candidate must be considered on an individual basis.

### Diagnostic algorithm

**Fig. 1:**
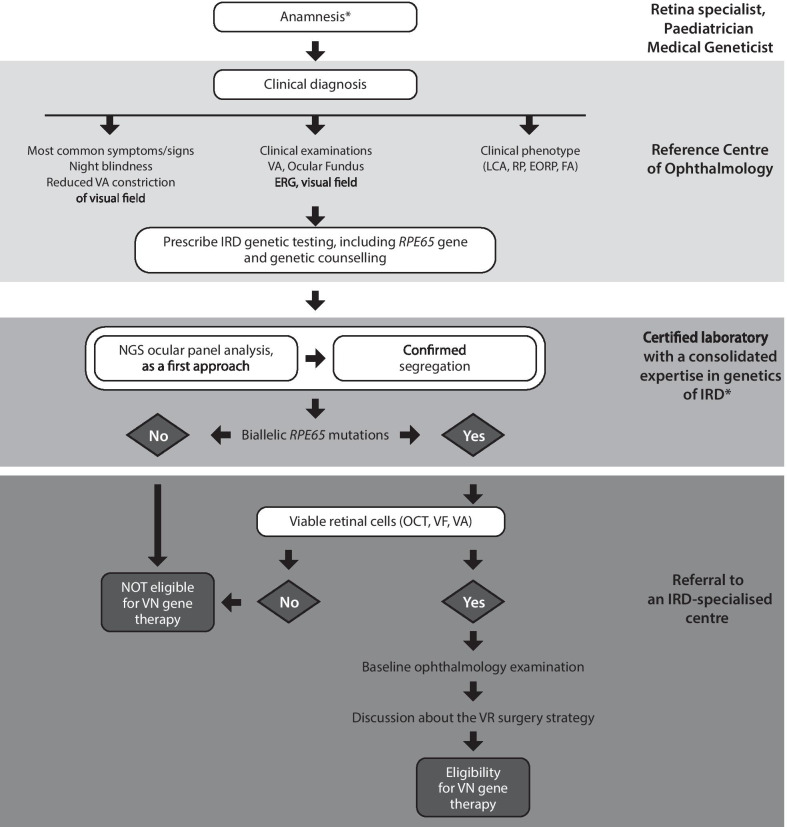
Clinical pathway algorithm to evaluate eligibility for voretigene neparvovec gene therapy. *Refer to Table 1 for further information. *FAF* fundus autofluorescence, *EORP* early-onset retinitis pigmentosa, *ERG* electroretinography, *FA* Fundus albipunctatus; *FST* full-field light sensitivity threshold; *IRD* inherited retinal disease, *LCA* Leber congenital amaurosis, *NGS* next-generation sequencing, *OCT* optical coherence tomography, *RP* retinitis pigmentosa, *RPE* retinal pigment epithelium, *VA* visual acuity, *GVF* Goldmann visual field, *VN* voretigene neparvovec, *VR* vitreoretinal

The consensus findings formed the basis for a diagnostic algorithm that was constructed by the steering committee (Fig. 1). The algorithm is designed to illustrate the patient pathway leading from the patient’s referral centre all the way to the retinal specialist centre. The primary intention was to develop a simple and linear algorithm to guide paediatricians and general ophthalmologists who are not experts in this area to arrive at the correct diagnosis of *RPE65*-associated IRD and make informed clinical decisions regarding eligibility for a gene therapy approach to *RPE65*-associated IRD. The summary algorithm will ultimately be developed into an expanded form by incorporating supplementary material that will detail the decision-making process presented in the summary algorithm.

## Discussion

The aim of this process was to provide practical guidance for the correct diagnosis of patients with *RPE65*-associated IRD, to identify the fundamental clinical tests to determine the correct diagnosis and to confirm patients eligible to receive treatment with voretigene neparvovec. The introduction of voretigene neparvovec as a gene supplementation therapy for *RPE65*-associated IRD is considered a milestone in the field of IRD. The pivotal phase 3 trial showed that a single sub-retinal injection of voretigene neparvovec is able to produce significant improvements in bilateral multi-luminance mobility test scores compared with controls at 1 year, with the beneficial effects maintained during the currently available 4 years of the planned 15 years of follow-up [48]. Furthermore, a recent evidence-based review by the UK National Institute for Health and Care Excellence (NICE) determined that the clinical benefits of voretigene neparvovec were important and represented a step change towards meeting the high unmet needs that have until now existed in RPE65-mediated IRD [49].

The heterogeneity and relative rarity of IRDs are further accentuated in *RPE65*-associated IRD [50], and present challenges for practising ophthalmologists who may have little exposure to orphan diseases. Although not specific to Italy alone, the limited number of qualified retinal specialist centres and a lack of access to the molecular resources necessary for diagnosis further compounds the issue of ensuring optimal treatment of *RPE65*-associated IRD. For example, a recent multinational survey by the European Vision Institute Clinical Research Network (EVICR.net) showed that genetic testing is not routinely performed or available in all centres and only about 50% of responding centres that see patients with IRDs have patients with confirmed biallelic *RPE65* mutations under care [51]. The survey also highlighted the current need for consensus and/or guidelines to inform standard-of-care in the current era of gene therapy. Therefore, it is becoming imperative that clinicians become more familiar with the recent advances in molecular diagnosis and that genetic laboratories are able to meet the level of competence required to provide accurate genotypic characterisation.

As the consequences of a diagnosis of *RPE65*-associated IRD are profound in terms of potential vision loss and impact on patients’ lives, optimising access to an expensive one-time therapy that may provide a lifetime beneficial effect is an important consideration. The panel members reached valuable consensus findings that direct attention to the relevant issues in identifying candidate patients for gene supplementation with voretigene neparvovec while clarifying matters that may be misunderstood or are of lesser relevance. The taking of a comprehensive anamnesis to establish the priorities and contribution of the key clinical manifestations is critical, and night blindness, reduced VA and narrowing of the visual field were identified as the three symptoms that are always present in *RPE65*-associated IRD. The clinical diagnosis, which is mainly LCA or EOSRD/EORP, needs to be established using fundamental clinical diagnostic tests (i.e. fundus examination, ERG, GVF examination and OCT), supported by FAF, FST and microperimetry for further investigation. Conducting a thorough assessment to ensure that the patient understands the implications of treatment and the necessity to comply with follow-up assessments post-treatment is also of the highest importance.

The role of clinical diagnosis is to reach a level of suspicion that justifies genetic testing in the presence of an IRD. There was a strong consensus for the value of targeted multi-gene NGS for diagnostic genetic testing, in preference to single-gene Sanger sequencing. Variants of uncertain significance (VUS) were identified as important in the diagnostic process and justify evaluation on a case-by-case basis rather than exclusion from consideration [40–46]. Correlations between mutation subtype and baseline visual function, response to voretigene neparvovec therapy, and adverse events were analysed in 29 patients in the phase 3 study [41]. No correlations between mutation subtype and baseline visual function or treatment response were identified, suggesting that the benefit/risk profile of voretigene neparvovec could not be predicted by mutation subtype [41].

The treatment of paediatric patients is a high priority in order to take full advantage of the opportunity for maintaining better VA and improving patients’ psychological well-being, relationships and family life. The safety and efficacy of voretigene neparvovec in children aged up to 4 years have not been established in clinical trials, although age in itself should not be a reason to exclude gene therapy. In the voretigene neparvovec clinical development programme (n = 41 patients), the average age of included patients was 17 years, ranging from 4 to 44 years of age. Of the 41 patients, 25 (61%) were paediatric patients under 18 years of age [28].

We identified a strong consensus that voretigene neparvovec should be initiated and administered by a vitreoretinal surgeon experienced in performing sub-retinal/macular surgery in association with a centre specialised in managing patients with IRD and where there is pharmacy capability for the handling of gene therapies. Surgeons and pharmacists from the treatment centres who meet these Risk Management Plan (RMP) criteria need to attend mandatory surgery/pharmacy education sessions in order to ensure the correct use of voretigene neparvovec and to minimise the risks associated with its administration and/or the administration procedure [28, 32].

Regarding the evaluation of viable retinal cells, based on the mechanism of action of voretigene neparvovec, the presence of sufficient viable retinal cells is considered necessary for therapeutic efficacy [52]. Although there are no universally shared criteria to unquestionably establish this viability, studies in the clinical development programme defined 101/102 and 301/302 approval trials [26, 28] defined sufficient viable retinal cells as an OCT showing more than a 100-μm thickness in an area of retina within the posterior pole and/or retina without atrophy or pigmentary degeneration within the posterior pole equal or larger than 3 disc areas and/or remaining GVF within 30 degrees of fixation as measured by a Goldmann isopter III-4e or equivalent. In this Delphi project, a consensus was reached that the most appropriate approach to assess viable cells, through a structural and functional evaluation, is to perform the clinical tests separately according to the sequence: OCT, followed by standard ophthalmological examinations including VA, posterior segment biomicroscopy, then GVF testing, microperimetry, colour picture, FAF and full-field ERG. The OCT should be the preferred and first-performed assessment to confirm eligibility for treatment. Indeed, thickness measurements on OCT served as an inclusion criterion in the phase 3 clinical programme [28], to estimate whether sufficient viable retinal cells were present for treatment. Of note, a correlation between OCT findings and therapeutic efficacy of voretigene neparvovec has not been reported [52].

This study brought together the expertise of a multidisciplinary panel representative of experts experienced in the management of IRDs in Italy and whose support was demonstrated by the high response rate achieved during the two Delphi rounds. Although the Delphi process is not designed to result in the highest level of evidence, it is an appropriate means of gaining consensus within a community, particularly when conducted by a fully representative panel of experts and supported by a comprehensive literature review of the subject. In the absence of definitive evidence-based literature and treatment guidelines on this rare disease, our Delphi process obtained valuable information that provides the basis for clinically useful guidance of a more focused and specific nature than the more general guidelines on the assessment and management of patients with IRD [53, 54]. Recommendations that set out the conditions to be met prior to performing treatment with, and to optimize outcomes with, voretigene neparvovec in Germany have been developed by the German Society of Ophthalmology, the German Retina Society e. V. and the Professional Association of German Ophthalmologists [55]. Their proposed diagnostic criteria to be fulfilled before treatment with voretigene neparvovec are largely in agreement with those in the present paper. However, we feel that our rigorous stepwise Delphi process consensus approach by an interdisciplinary panel of experts, supported by an extensive review of the literature, strengthens the recommendations presented here. The statements presented in the Round 1 questionnaire of our study were carefully constructed to avoid ambiguity, validated before distribution, and appropriately modified for Round 2 based on the Round 1 responses. Analysis of the Round 1 and Round 2 responses was by an expert steering committee with considerable experience in the field of IRDs, and a consensus was achieved across a wide range of issues.

## Conclusions

To summarise, this paper provides robust, evidence-based and consensus-driven guidelines that can be used by ophthalmologists and paediatricians to arrive at the correct diagnosis of *RPE65*-associated IRD and to make informed clinical decisions about eligibility for gene supplementation with voretigene neparvovec. This is particularly important in a rare condition, such as *RPE65*-associated IRD, where progressive visual impairment has a distressing impact on many aspects of patients’ lives. Although voretigene neparvovec makes available to clinicians a treatment with the potential to prevent blindness and provide a life-long beneficial effect for many patients with *RPE65*-associated IRD, its safe and effective use requires expertise across a multidisciplinary team that includes ophthalmologists, geneticists, surgeons, and patient support services.

The process of securing consensus among representative experts in the management of patients with IRDs has facilitated the development of a practical diagnostic algorithm to guide paediatricians and general ophthalmologists encountering IRDs in clinical practice. Although there are currently unmet needs regarding genetic counselling in *RPE65*- associated IRD, the limited availability of genetic testing and the long turnaround time in clinical practice, we consider that the information here presented can serve as a framework of care for the optimal treatment of these patients.

## Supplementary Information

**Additional file 1: Table S1.** Clinical manifestations of *RPE65*-associated IRD. Provides an overview of the consensus during the Round 1 and Round 2 questionnaires on statements relating to the clinical manifestations of *RPE65*.

**Additional file 2: Table S2.** Viable retinal cells: structure and function. Provides an overview of the consensus during the Round 1 and Round 2 questionnaires on statements relating to the structure and function of retinal cells.

**Additional file 3: Table S3.** Genetic testing. Provides an overview of the consensus during the Round 1 and Round 2 questionnaires on statements relating to genetic testing for IRD.

**Additional file 4: Table S4.** Patient eligibility and prioritisation. Provides an overview of the consensus during the Round 1 and Round 2 questionnaires on statements relating to patient identification and prioritisation of treatment.

**Additional file 5: Table S5.** Assessment of surgical strategy. Provides an overview of the consensus during the Round 1 and Round 2 questionnaires on statements relating to surgery for IRD.

**Additional file 6: Appendix.** Members of the panel of experts. List of all members of the panel of experts who participated in the Delphi consensus process.

## Data Availability

All data generated or analysed during this study are included in this published article [and its supplementary information files].

## Abbreviations

cGH: Comparative genomic hybridisation
FST: Full-field light sensitivity threshold
EORP: Early-onset retinitis pigmentosa
EOSRD: Early-onset severe retinal dystrophy
ERG: Electroretinography
FA: Fundus albipunctatus
FAF: Fundus autofluorescence
GVF: Goldmann visual field
IRD: INHERITED retinal disease
LCA: Leber congenital amaurosis
MLMT: Multiluminance mobility test
MLPA: Multiplex ligation-dependent probe amplification
MRI: Magnetic resonance imaging
NGS: Next-generation sequencing
OCT: Optical coherence tomography
RMP: Risk Management Plan
RP: Retinitis pigmentosa
RPE: Retinal pigment epithelium
SIGU: Società Italiana di Genetica Umana
VA: Visual acuity
VN: Voretigene neparvovec
VR: Vitreoretinal
VUS: Variants of uncertain significance
