# Supplementary material for: RPE65-associated inherited retinal diseases: consensus recommendations for eligibility to gene therapy
Source: Orphanet J Rare Dis. 2021 Jun 4;16:257. doi: 10.1186/s13023-021-01868-4 (PMC8176684; doi:10.1186/s13023-021-01868-4)
Supplement: Supplementary file 1 — Additional file 1: Table S1. Clinical manifestations of RPE65-associated IRD. Provides an overview of the consensus during the Round 1 and Round 2 questionnaires on statements relating to the clinical manifestations of RPE65. [file 13023_2021_1868_MOESM1_ESM.pdf]

Table S1. Clinical manifestations of RPE65-associated IRD. Level of agreement after completion of Rounds 1 and 2. A cut-off of 70 (Agree and Strongly agree on the 5-point Likert scale) was defined as consensus. Statements not included in a survey Round are marked with "–" in the relevant column. Items where the general mean of the sample deviated significantly from the responses stratified by medical speciality are marked with an asterisk.

No.	Statement	Degree of consensus, %	
		**First round**	**Second round**
1.1	RPE65-associated inherited retinal diseases affect mainly paediatric patients	–	73.0
1.2	First clinical manifestations are observed shortly after birth or in the first few years of life (during childhood or adolescence)	–	94.6
1.3	The clinical diagnosis is mainly Leber congenital amaurosis (LCA) or early-onset severe retinal dystrophy/early-onset retinitis pigmentosa	–	91.7
1.4	Fundus albipunctatus (FA) is a rare autosomal recessive form of stationary night blindness and can be associated with *RPE65* biallelic mutations	–	75.0
1.5	Mutations in the *RPE65* gene account for about 6% to 16% of LCA diagnosis	–	80.6*
1.6	Mutations in the *RPE65* gene account for 2% of retinitis pigmentosa diagnosis	–	80.6*
2	There is no direct correlation between genotype and phenotype in *RPE65* mutation-associated inherited retinal disease	67.9	–
2	There is a direct correlation between genotype and phenotype in *RPE65* mutation-associated inherited retinal disease	–	22.2*
3	Nyctalopia (night blindness) due to decreased sensitivity to light is the most common symptom in patients with *RPE65* mutation-associated inherited retinal disease	69.0	72.2*
4	Nystagmus (rapid, rhythmic, and involuntary eye movement), is a common symptom of patients with *RPE65* mutation-associated inherited retinal disease	72.4	86.1
5	In *RPE65* mutation-associated inherited retinal disease:		
5.1	Not all patients with *RPE65* mutation-associated inherited retinal disease have significantly reduced visual acuity	48.3	–
5.1	Visual acuity may vary and is heterogeneous between patients at onset	–	82.9*
5.2	Visual acuity may show stable acuities or progressive decline with age	–	82.9
5.3	Most patients have a visual acuity of 0.5 LogMar (0.32 decimal) or worse	–	60.6*
5.4	Some patients have a visual acuity better than 0.5 LogMar (0.32 decimal)	–	51.5*
5.5	Patients with RPE65-associated Fundus albipunctatus (a form of congenital night-blindness disorder) have a good or entirely normal (1 decimal) visual acuity and preserved visual fields into adulthood	–	55.6*

5.6	In most individuals, visual acuity is near-normal and fairly stable at young ages, begins to decrease gradually around the ages 15 to 20 years of age, and then undergoes rapid acceleration of the rate of visual acuity loss after the age of 20	–	47.1*
5.7	Progression of visual field constriction begins gradually from the first decade of life	–	54.3*
5.8	Visual field decline is more linear and gradual than visual acuity loss	–	41.2*
5.9	The limiting factor for improving visual acuity post-treatment may not be the efficiency of RPE65-mediated gene therapy, but rather the timing of therapy, with treatment very early in childhood providing optimal recovery of visual acuity	–	82.9
6	When assessing a patient with suspected *RPE65* mutation-associated inherited retinal disease, the anamnesis (medical history) must include:	–	
6.1	General ophthalmologic history	96.6	97.3
6.2	Symptoms at onset	96.6	100.0
6.3	Age at symptom onset	96.6	100.0
6.4	Pedigree	96.6	100.0
6.5	Inquiry about consanguinity	96.4	91.9
6.6	Existence of other affected family members	93.1	100.0
6.7	Signs of disease progression	93.1	97.3
6.8	Previous/current therapy for ocular diseases	79.3	91.9
7	Which of these other topics listed below should be included in the anamnesis?	–	
7.1	Other previous visits to a general ophthalmologist or to a retina specialist	–	81.1
7.2	Previous clinical assessment for vision (MRI, OCT, ERG, FAF)	–	94.6
7.3	Presence or not of neurological or extra-ocular symptoms	–	91.9
7.4	General pharmacologic history and ongoing medical treatments	–	83.8
7.5	History of infectious diseases	–	89.2
7.6	Other diseases	–	91.9
7.7	Patient expectations	–	86.5
7.8	Professional activities and lifestyle (for adult patients)	–	78.4
8	The symptoms that I consider most indicative of *RPE65* mutation-associated inherited retinal disease include:		
8.1	Photophobia	50.0	63.9
8.2	Visual field narrowing	75.9	88.6
8.3	Reduction in visual acuity	79.3	86.1
8.4	Nystagmus (rapid involuntary rhythmic eye movement)	75.9	94.4
8.5	Night blindness	86.2	100.0
8.6	Abnormal fixational eye movements or retinal fixation location	55.2	74.3*
9	What other symptoms should be considered highly indicative of *RPE65* mutation-associated inherited retinal disease?	–	
9.1	None, the symptoms mentioned in the previous item are sufficient	–	20.7
9.2	Franceschetti’s oculo-digital sign (eye rubbing)	–	57.6
9.3	Reduced colour vision/No colour vision	–	39.4

9.4	Reduced physiological fundus autofluorescence	–	57.6*
9.5	Thinning of retinal thickness at the OCT examination	–	54.5*
9.6	Peripheral photopsia	–	25.0
9.7	Reduced contrast sensitivity	–	28.1
9.8	Poor correlation between visual impairment and anatomical retinal damage	–	57.6
10	Fundamental clinical diagnostic tests (i.e., tests with the highest diagnostic value) are:		
10.1	Fundus examination	93.1	100.0
10.2	Visual field examination	82.8	97.2
10.3	Full-field electroretinogram (ERG)	93.1	100.0
10.4	Optical Coherence Tomography (OCT)	86.2	94.4
11	Tests for further investigation should include:		
11.1	Full-field light sensitivity threshold testing (D-FST)	67.9	61.8*
11.2	Microperimetry	60.7	58.8
11.3	Fundus autofluorescence (FAF)	82.1	79.4
11.4	Multifocal electroretinogram	48.3	48.6
11.5	Chromatic pupillometry	44.4	55.9
11.6	Visual evoked potentials	42.9	50.0
