# Supplementary material for: RPE65-associated inherited retinal diseases: consensus recommendations for eligibility to gene therapy
Source: Orphanet J Rare Dis. 2021 Jun 4;16:257. doi: 10.1186/s13023-021-01868-4 (PMC8176684; doi:10.1186/s13023-021-01868-4)
Supplement: Supplementary file 2 — Additional file 2: Table S2. Viable retinal cells: structure and function. Provides an overview of the consensus during the Round 1 and Round 2 questionnaires on statements relating to the structure and function of retinal cells. [file 13023_2021_1868_MOESM2_ESM.pdf]

Table S2. Viable retinal cells: structure and function. Level of agreement after completion of Rounds 1 and 2. A cut-off of 70 (Agree and Strongly agree on the 5-point Likert scale) was defined as consensus. Statements not included in a survey Round are marked with "–" in the relevant column. Items where the general mean of the sample deviated significantly from the responses stratified by medical speciality are marked with an asterisk.

No.	Statement	Degree of consensus, %	
		First round	Second round
13	Residual viable retinal cells and their functioning should be assessed with:		
13.1	Optical Coherence Tomography (OCT)	78.6	97.1
13.2	Visual acuity	57.1	76.5*
13.3	Goldmann visual field (GVF)	51.9	73.5*
13.4	Full-field electroretinogram (ERG)	71.4	85.7
13.5	Fundus autofluorescence (FAF)	60.7	90.9
13.6	Microperimetry (MP)	60.7	79.4
14	The most appropriate approach to assess viable cells is to perform the clinical tests separately according to this sequence:	–	
14.1	First level: OCT	–	75.8
14.2	Second level: standard ophthalmological examinations including visual acuity, anterior & posterior segment by microscopy	–	63.6*
14.3	Third level: GVP, MP, colour picture, FAF, ERG	–	72.7
15	OCT is the most suitable but not the only test for measuring the remaining vital retinal cells	75.0	79.4
