# Supplementary material for: RPE65-associated inherited retinal diseases: consensus recommendations for eligibility to gene therapy
Source: Orphanet J Rare Dis. 2021 Jun 4;16:257. doi: 10.1186/s13023-021-01868-4 (PMC8176684; doi:10.1186/s13023-021-01868-4)
Supplement: Supplementary file 3 — Additional file 3: Table S3. Genetic testing. Provides an overview of the consensus during the Round 1 and Round 2 questionnaires on statements relating to genetic testing for IRD. [file 13023_2021_1868_MOESM3_ESM.pdf]

Table S3. Genetic testing. Level of agreement after completion of Rounds 1 and 2. A cut-off of 70 (Agree and Strongly agree on the 5-point Likert scale) was defined as consensus. Statements not included in a survey Round are marked with "–" in the relevant column. Items where the general mean of the sample deviated significantly from the responses stratified by medical speciality are marked with an asterisk.

No.	Statement	Degree of consensus, %	
		**First round**	**Second round**
17	Genetic testing must be prescribed and performed in all cases where there is suspicion of an inherited retinopathy	96.7	95.0
18	For routine diagnosis, genetic tests must be based on:		
18.1	Sanger sequencing of the *RPE65* gene only	23.1	11.4
18.2	A targeted, multi-gene Next-generation sequencing (NGS) panel, including retinopathy-associated genes	96.7	92.3
18.3	As a first test, an extended NGS analysis (clinical exome or whole-exome sequencing)	38.5	40.5
19	Genetic testing for diagnosis must be carried out by certified laboratories	100.0	95.0
20	The certification of a genetic diagnostic laboratory is defined by the following criteria:		
20.1	ISO certification	86.7	84.2*
20.2	Analyse >100 cases per year for genetic diagnosis and document a highly significant number of confirmed genetic diagnosis cases	83.3	84.6
20.3	Being part of a network with medical geneticists and inherited retinal diseases specialists from other national and international centres	86.7	94.9
21	The certified laboratories conducting genetic testing for inherited retinal disease diagnosis must:		
21.1	Have qualified geneticists with consolidated expertise in the genetics of hereditary retinal dystrophies	93.3	95.0
21.2	Have standardised internal molecular analysis protocols	100.0	97.5
21.5	Perform genetic counselling before and after testing	90.0	100.0
21.6	Be part of a national diagnostic laboratories network and/or Genetic Scientific Society (e.g., SIGU)	80.0	85.0
21.7	Rely on a complete multidisciplinary team (geneticists, retina specialist, molecular biologists, technicians, bioinformatician, genetic counsellor) already familiar with IRDs molecular diagnosis	–	97.4
21.8	Be able to perform MLPA analysis	–	87.5
21.9	Be able to perform both Sanger and multi-gene NGS tests	–	100.0
21.10	Be able to perform in silico analysis	–	90.0
21.11	Be able to perform in vitro protein functional assessment	–	59.0
21.12	Participate in inherited retinal disease national/international registries	–	87.5
22	A qualified geneticist is defined as a geneticist with:	–	
22.1	Consolidated expertise in the genetics of hereditary retinal dystrophies	–	95.0

22.2	Updated knowledge of the state-of-the-art and proven track record in the field of genetics of IRDs	–	95.0
22.3	Relevant published literature in the field	–	75.0
22.4	Proactive interactions and collaborations with international counterparts as part of multicentre consortia	–	84.6*
23	Active networking with national and international counterparts is particularly important for a qualified geneticist:	–	
23.1	In the case of rare diseases with high genetic heterogeneity like RPE65-associated Inherited retinal disease	–	95.0
23.2	To exchange knowledge and expertise with other geneticists and IRD specialists	–	92.5
23.3	To collect evidence that may strengthen suspicions about the causative role of VUS	–	95.0
24	Analysis of *RPE65* gene mutation segregation:		
24.1	Is obligatory when assessing any patient with suspected *RPE65* mutation-associated inherited retinal disease	76.7	70.0
24.2	Is obligatory when the identified variants are pathogenic, likely pathogenic and VUS (uncertain significance)	90.0	95.0
24.3	Is obligatory in case of compound heterozygous mutations (when two different *RPE65* variants are identified by genetic testing)	96.7	95.0
24.4	Is not obligatory in case of homozygous mutation (when a mutation of *RPE65* is identified in the absence of a wildtype allele)	20.0	42.5
24.5	Is highly recommended when a homozygous mutation is suspected, because this could be confused with a loss of heterozygosity	86.7	87.5
24.6	Should be performed in all patients, including cases in which a variant is identified at a homozygous state, because it helps to fully define the patient's genotype	–	87.5
24.7	Would be useful for patients with monoallelic complex variants in cis (i.e., and are not biallelic for the identified variants), in whom it may be worth employing additional screening methods to look for larger CNV or in non-coding regions of *RPE65* gene	–	97.4
24.8	Should not represent an exclusion criterion for a patient's eligibility for an *RPE65* gene therapy-based treatment, since for some patients (e.g., cases of adopted patients, cases of non-paternity, cases of deceased parent/s) it may not be feasible to perform segregation analysis	–	87.2
25	Extending the investigation of *RPE65* mutation segregation to additional family members can provide more support for eligibility in patients with *RPE65* Variants of Uncertain Significance (VUS)	86.7	95.0
26	In-depth discussion between geneticists and IRD (inherited retinal disease) specialists is crucial to assess possible correlations between *RPE65* Variants of Uncertain Significance (VUS) and clinical manifestations for patients	90.0	95.0
27	Patients with *RPE65* Variants of Uncertain Significance may be candidates for Voretigene Neparvovec therapy if they have:		
27.1	One confirmed pathogenic or likely pathogenic variant and one Variant of Uncertain Significance (VUS)	56.7	70.0

27.2	Two Variants of Uncertain Significance (VUS) confirmed by extended segregation to more family members and accompanied by clinical evidence consistent with an inherited retinal disease	40.0	75.0
27.3	Two Variants of Uncertain Significance (VUS) confirmed by segregation and reported in the literature, accompanied by clinical evidence consistent with an inherited retinal disease	50.0	72.5
27.4	Two Variants of Uncertain Significance (VUS) with predicted pathological impact confirmed by an in silico predictive algorithm and by extended segregation	34.5	57.5
27.5	Two Variants of Uncertain Significance (VUS) with predicted pathological impact by *in silico* and confirmed segregation that are accompanied by clinical evidence consistent with an inherited retinal disease	63.3	77.5
27.6	Two biallelic Variants of Uncertain Significance (VUS) with pathogenicity confirmed by *in vitro* protein functional assessment accompanied by clinical evidence consistent with an inherited retinal disease	60.0	80.0*
28	Compared to the Sanger method, Next-generation Sequencing provides information that allows a more accurate genetic diagnosis of *RPE65* mutation-associated inherited retinal disease	62.1	64.1*
29	The Sanger validation of the *RPE65* variants identified by NGS-based approaches is essential and indispensable even for variants that had very good coverage in the NGS analysis		59.0*
30	The technical time required to conduct genetic testing of the *RPE65* gene with NGS is two months	26.7	–
30	The technical time required to conduct genetic testing of *RPE65* with NGS including bioinformatic data analysis and validation is:	–	
30.1	Between 3 and 6 months	–	67.5*
30.2	Between 6 and 8 months	–	41.0
30.3	Between 8 and 12 months	–	25.6
31	Analysis of blood samples provides higher quality results than analysis on saliva samples		71.8
32	Simultaneous collection of (i) patient samples for mutations and (ii) parental samples for segregation reduces the time to genetic diagnosis of *RPE65* mutation-associated inherited retinal disease	66.7	67.5*
