# Supplementary material for: RPE65-associated inherited retinal diseases: consensus recommendations for eligibility to gene therapy
Source: Orphanet J Rare Dis. 2021 Jun 4;16:257. doi: 10.1186/s13023-021-01868-4 (PMC8176684; doi:10.1186/s13023-021-01868-4)
Supplement: Supplementary file 4 — Additional file 4: Table S4. Patient eligibility and prioritisation. Provides an overview of the consensus during the Round 1 and Round 2 questionnaires on statements relating to patient identification and prioritisation of treatment. [file 13023_2021_1868_MOESM4_ESM.pdf]

Table S4. Patient eligibility and prioritisation. Level of agreement after completion of Rounds 1 and 2. A cut-off of 70 (Agree and Strongly agree on the 5-point Likert scale) was defined as consensus. Statements not included in a survey Round are marked with "–" in the relevant column. Items where the general mean of the sample deviated significantly from the responses stratified by medical speciality are marked with an asterisk.

**No.**	**Statement**	**Degree of consensus, %**	
		**First round**	**Second round**
33	Progression alone is not a prognostic criterion	48.3	–
34	Disease progression rate for RPE65-associated inherited retinal disease can be defined by one or more parameters including:	–	
34.1	Visual field loss	–	97.2
34.2	Visual acuity deterioration	–	94.4
34.3	Viable retinal cells by OCT evaluation	–	88.9
35	The following criteria are useful for "prioritising" eligible patients to receive treatment with Voretigene Neparvovec		
35.1	Age	82.8	94.6
35.2	Disease progression rate	79.3	83.8
35.3	Visual acuity status	75.9	66.7
35.4	Amount of viable retinal cells	89.7	94.6
35.5	Confirmed *RPE65* mutations pathogenicity (category 4 and 5, likely pathogenic and pathogenic)	96.6	94.4
36	Other aspects to consider in prioritisation are:	–	
36.1	Psychological assessment and the patient's attitude toward treatment	–	86.5
36.2	Early treatment for paediatric patients	–	97.3
36.3	Early treatment for patients with good visual acuity (higher than 05 LogMar, 032 decimal) to prevent progression	–	77.1
36.4	Early treatment for patients with low visual acuity (05 LogMar, 032 decimal or worse) to preserve the remaining vision	–	77.1
36.5	Handheld instruments use (OCT, retinal imaging, ERG) in case of children less than 3 years old	–	73.5
36.6	Patient's compliance with follow-up assessments post-treatment	–	88.9
37	A paediatric patient can be a candidate for gene therapy with Voretigene Neparvovec starting from the age of 3 years	72.4	66.7
38	It is important to give high priority to the treatment of paediatric patients, in order to provide them with the opportunity for maintaining a better visual acuity and enjoying a normal social growth	89.3	89.2
39	Age is not a criterion for exclusion from gene therapy in patients with age > 3 years	89.7	86.5
