# Supplementary material for: RPE65-associated inherited retinal diseases: consensus recommendations for eligibility to gene therapy
Source: Orphanet J Rare Dis. 2021 Jun 4;16:257. doi: 10.1186/s13023-021-01868-4 (PMC8176684; doi:10.1186/s13023-021-01868-4)
Supplement: Supplementary file 5 — Additional file 5: Table S5. Assessment of surgical strategy. Provides an overview of the consensus during the Round 1 and Round 2 questionnaires on statements relating to surgery for IRD. [file 13023_2021_1868_MOESM5_ESM.pdf]

Table S5. Assessment of surgical strategy. Level of agreement after completion of Rounds 1 and 2. A cut-off of 70 (Agree and Strongly agree on the 5-point Likert scale) was defined as consensus. Statements not included in a survey Round are marked with "–" in the relevant column. Items where the general mean of the sample deviated significantly from the responses stratified by medical speciality are marked with an asterisk.

No.	Statement	Degree of consensus, %	
		**First round**	**Second round**
40	Patients with suspected RPE65-associated IRD must be referred to a clinical centre specialised in inherited retinal diseases	100.0	100.0
41	The vitreoretinal surgeon who performs the subretinal injection must be affiliated to an Inherited Retinal Disease Centre certified for the use of gene therapy with Voretigene Neparvovec	86.2	91.9
42	The vitreoretinal surgeons must have surgical experience with patients with inherited retinal diseases	86.2	–
42	The vitreoretinal surgeons must have surgical experience with paediatric and adult patients with inherited retinal diseases	–	89.2
43	Inherited Retinal Diseases retina specialist must have in-depth discussions with the certified vitreoretinal surgeon to:		
43.1	Confirm the best surgery strategy for each patient candidate to treatment with Voretigene Neparvovec	92.6	97.1
43.2	Assess vitrectomy surgery and subretinal injection risks	85.7	91.7
44	The following topics must be discussed with the certified surgeon to confirm patient eligibility for surgery and to assess surgery risks:		
44.1	Patient age	75.0	83.8
44.2	Retinal thickness	82.1	94.6
44.3	Other eye disorders	82.1	89.2*
44.4	Injection site	71.4	89.2
45	What other topics must be discussed with the certified surgeon to confirm patient eligibility for surgery and to assess surgery risks:	–	
45.1	A paediatric hospital setting for children-centred care (3-6 years old) delivery and proper management of paediatric anesthesiology procedures	–	91.2
45.2	Presence of cataract as an exclusion criteria	–	42.4*
45.3	Eligibility for general anaesthesia	–	83.3
45.4	Vitrectomy technique: three-port posterior vitrectomy 25G and mandatory posterior vitreous detachment	–	63.3*
45.5	Vitrectomy technique: possibility to use vitreous staining such as triamcinolone	–	58.6*
45.6	Evaluate surgery risk/treatment benefit ratio	–	88.2
45.7	Anamnesis: clinical history, genetic diagnosis, expectations of the patient regarding clinical outcomes	–	90.9
