# Supplementary material for: RPE65-associated inherited retinal diseases: consensus recommendations for eligibility to gene therapy
Source: Orphanet J Rare Dis. 2021 Jun 4;16:257. doi: 10.1186/s13023-021-01868-4 (PMC8176684; doi:10.1186/s13023-021-01868-4)
Supplement: Supplementary file 6 — Additional file 6: Appendix. Members of the panel of experts. List of all members of the panel of experts who participated in the Delphi consensus process. [file 13023_2021_1868_MOESM6_ESM.pdf]

Appendix: Members of the Panel of Experts.

Validation Panel (n = 4): Brunella Franco, Dario Giorgio, Valentina Di Iorio, Raffaella Brunetti Pierri.

Survey Panel

Ophthalmologists (n = 13): Giancarlo Iarossi, Sabrina Signorini, Chiara Bertone, Maria Pia Manitto, Francesco Parmeggiani, Giacomo Bacci, Anna Paola Salvetti, Marta Oldani, Giovanni Staurenghi, Leonardo Colombo, Benedetto Falsini, Vittoria Murro, Dario Mucciolo.

Geneticists (n = 30): Cristiana Marchese, Enza Maria Valente, Sara Bargiacchi, Francesca Rivieri, Gaia Roversi, Matteo Bertelli, Giulia Guerri, Marianthi Karali, Alberto Auricchio, Nicola Brunetti Pierri, Teresa Divizia, Ilaria Donati, Marco Seri, Claudio Graziano, Vincenzo Nigro, Paolo Gasparini, Irene De Rienzo, Enrico Grosso, Francesca Torricelli, Luca Maria Rocchetti, Achille Iolascon, Angelo Selicorni, Maurizio Genuardi, Corrado Romano, Andrea Angius, Alfredo Brusco, Valeria Marigo, Enrico Maria Surace, Luisa Candita, Chiara Pescucci.

Vitreoretinal surgeons (n = 8): Stanislao Rizzo, Tomaso Caporossi, Francesco Barca, Fabrizio Giansanti, Marzio Chizzolini, Daniele Tognetto, Matteo Cereda, Grazia Pertile.
